# Mediation effects of mean Hounsfield unit on relationship between hemoglobin and expansion of intracerebral hemorrhage

**DOI:** 10.1038/s41598-021-96790-x

**Published:** 2021-08-26

**Authors:** Yong Soo Kim, Han-Gil Jeong, Hee-Yun Chae, Beom Joon Kim, Jihoon Kang, Jun Yup Kim, Tackeun Kim, Jae Seung Bang, Hee-Joon Bae, Chang Wan Oh, Moon-Ku Han

**Affiliations:** 1 Department of Neurology, Seoul National University Bundang Hospital, 300 Gumi-dong, Bundang-gu, Seongnam, Gyeonggi-do 463–707 Republic of Korea; 2 Department of Neurosurgery, Seoul National University Bundang Hospital, Seongnam, Republic of Korea; 3 Department of Neurology, Chungbuk National University Hospital, Cheongju, Republic of Korea

**Keywords:** Stroke, Brain

## Abstract

Low hemoglobin levels are known to be associated with hematoma expansion (HE) and poor functional outcome in patients with intracerebral hemorrhage (ICH). However, it is not yet known whether low hemoglobin itself causes HE directly or is merely a confounder. Thus, we investigated the mediation effect of the mean Hounsfield unit (HU) of hematoma on the relationship between low hemoglobin and expansion of ICH. Overall, 232 consecutive patients with ICH who underwent non-contrast computed tomography (NCCT) within 12 h since onset were included. The mean HU and hematoma volume on NCCT were investigated using semi-automated planimetry. HE was defined as an increase in hematoma volume > 33% or 6 mL. The respective associations among the hemoglobin level, mean HU, and HE were analyzed using multivariable regression analysis, adjusting for age, sex, and known HE predictors. Mediation analysis was performed to examine the potential causal association among the three. HE occurred in 34.5% of patients; hemoglobin levels were inversely associated with HE occurrence (adjusted odds ratio, 0.90; *p* = 0.03). The mean HU of the hematoma was lower in patients with HE than in patients without HE (58.5 ± 3.3 vs. 56.8 ± 3.0; *p* < 0.01). Hemoglobin levels on admission were linearly related to the mean HU (adjusted β, 0.33; *p* < 0.01) after adjusting for known HE predictors (time from onset to CT, antithrombotic use, hematoma volume). Causal mediation analysis showed a significant mediation effect of the mean HU on the association between hemoglobin levels and HE (*p* = 0.04). The proportion of indirect effect through the mean HU among the total effect was 19% (*p* = 0.05). The mediation effect became nonsignificant in the when the multivariable model was adjusted with additional covariates (baseline systolic blood pressure and hematoma location). The mean HU of the hematoma mediated the association between hemoglobin levels and HE occurrence. Therefore, the mean HU of the hematoma may be a potential marker of impaired hemostasis in patients with ICH.

## Introduction

Hematoma expansion (HE) is the cause of early neurologic deterioration, leading to poor functional outcomes after intracerebral hemorrhage (ICH). Various clinical and imaging predictors of HE have been identified. Modification of these factors, including intensive blood pressure control and hemostatic therapies, showed potential in limiting HE but failed to improve clinical outcomes^1,2^. Thus, a more personalized approach to preventing HE is warranted based on risk profiles. The pathophysiology of HE also needs to be further elucidated to develop novel treatment strategies.

Patients with ICH having lower hemoglobin levels are known to have poor functional outcomes^3^. Lower hemoglobin levels may represent patients with more comorbidities^4^. However, erythrocytes have been known to have significant roles in hemostasis through dynamic interactions with platelets and fibrin networks^5^. A recent study has demonstrated that the effect of lower hemoglobin levels on functional outcomes is significantly mediated via the occurrence of HE^4^. However, whether low hemoglobin itself contributes to HE or is merely a confounder of the vulnerability of hematoma remains unknown.

Among the predictive markers of HE on non-contrast computed tomography (NCCT), the mean Hounsfield unit (HU) of hematoma may reflect the process of clot contraction and local hemostasis^6^. Thus, analyzing the relationship among hemoglobin levels, the mean HU of hematoma, and HE may help elucidate HE mechanisms in patients with low hemoglobin^6^. We aimed to test the hypothesis that the mean HU of hematoma mediates the effects of low hemoglobin on HE in patients with ICH.

## Methods

### Patient selection

**Figure 1:**
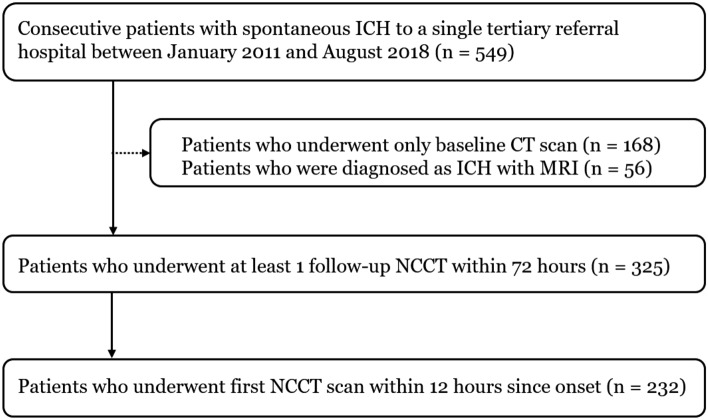
Flow diagram of patient selection. ICH, Intracerebral hemorrhage; CT, computed tomography; MRI, magnetic resonance imaging; NCCT, non-contrast computed tomography.

A total of 549 consecutive patients with acute ICH admitted to a neurology department of a single tertiary referral hospital between January 2011 and August 2018 were included in this retrospective study (Fig. 1). The inclusion criteria were as follows: (1) underwent at least one follow-up NCCT (n = 325) and (2) last known normal time (LNT) to first NCCT scan within 12 h (n = 232). The acute treatment strategies for the patients with ICH followed the guideline of American Heart Association/American Stroke Association guidelines for the management of spontaneous ICH. However, intravenous tranexamic acid was used selectively by physician’s discretion between 2017 and 2018. This study was approved by the institutional review board of Seoul National University Bundang Hospital (approval number: B-2011/648-105). The written informed consent form was waived by the institutional review board due to the retrospective nature of the study. All study methods were carried out in accordance with relevant ethical guidelines and regulations.

### Clinical data collection

Baseline demographic and clinical information of patients, including age; sex; baseline systolic blood pressure; LNT; arrival time to the hospital; National Institute of Health Stroke Scale score (NIHSS) on admission; and previous medical histories, such as the presence of hypertension, diabetes mellitus, hyperlipidemia, atrial fibrillation, antiplatelet use, and anticoagulant use, was collected from our stroke registry. Laboratory information included the hemoglobin level, hematocrit, white blood cell count, platelet count, prothrombin time, creatinine level, and glucose in the first blood sample upon arrival at the emergency room.

### Imaging data collection and definition

NCCT was performed as per our standard protocol with 64- or 128-slice CT scanners (Brilliance 64 and iCT; Phillips Medical Systems, Best, The Netherlands), using the axial technique adjusted for the following parameters: 120 kVp, 190–250 mA, and 5-mm slice thickness reconstruction.

Baseline NCCT scans of 232 patients were initially reviewed by two stroke neurologists (KYS, CHY) independently, blinded to clinical data and existence of hematoma growth, to determine the ICH location (basal ganglia, thalamus, lobar, posterior fossa), presence of intraventricular hemorrhage, and NCCT markers known to predict HE. NCCT markers included heterogeneous density, the black hole sign, blend sign, irregular shape, island sign, and satellite sign^7–11^. Discrepancies between the two readers were adjudicated by joint discussion until consensus was reached.

**Figure 2:**
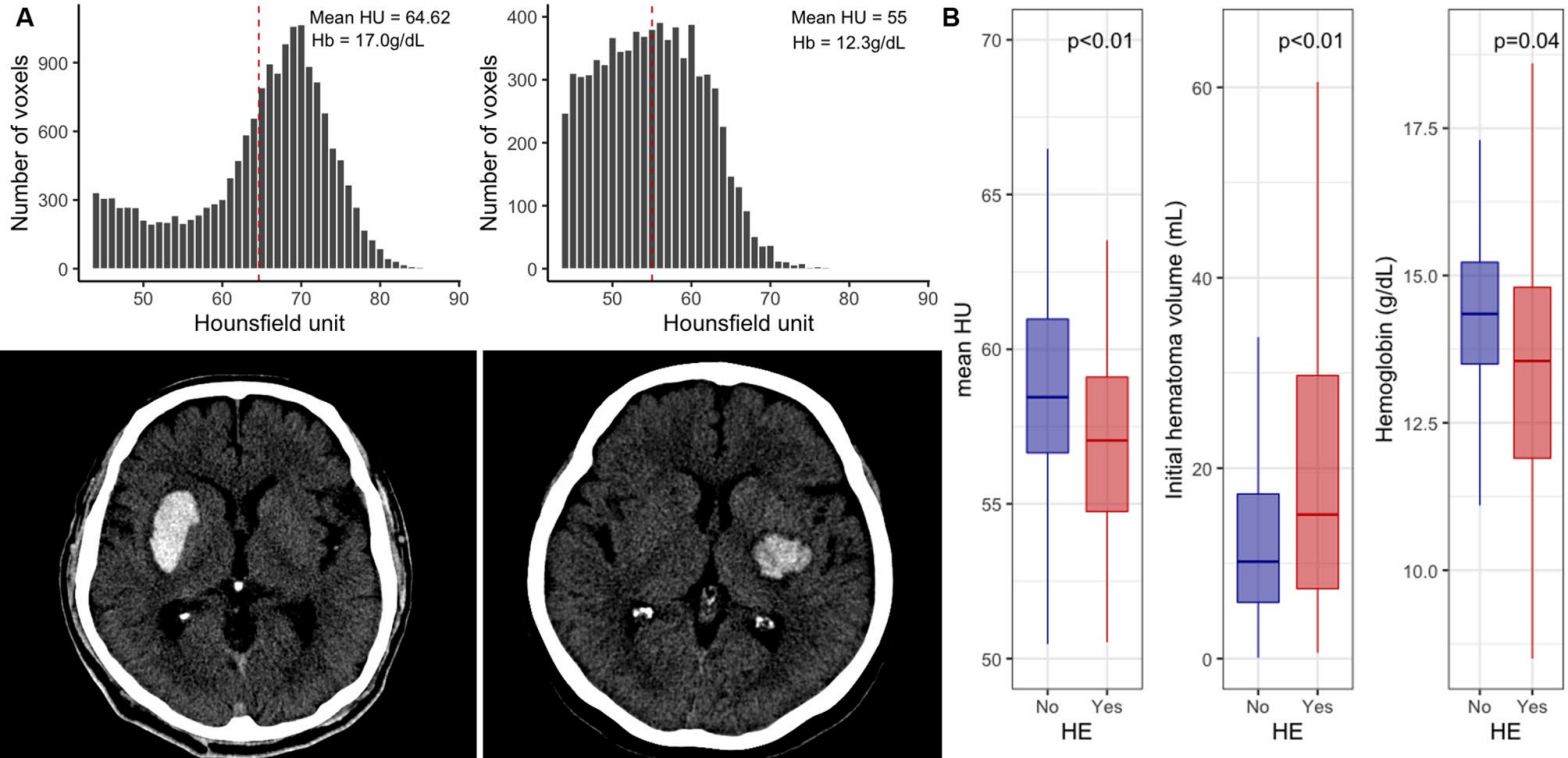
Representative cases and difference in mean HU, hematoma volume, hemoglobin according to hematoma expansion. (**A**) Two representative cases with hematoma expansion (HE) (left) and without HE (right). (**B**) Association among the mean Hounsfield unit, baseline hematoma volume, and hemoglobin with HE. HU, Hounsfield unit; Hb, Hemoglobin.

After visual inspection of baseline CT scans, all scans taken within 72 h since arrival (n = 493) were analyzed to measure the volume and density (Hounsfield unit, HU) of the hematoma using the Analyze 14.0 software (Analyze Direct, Inc., Overland Park, KS). When multiple CT scans were taken within 72 h, the first CT scan that meets the definition of HE was selected for the analysis. After segmentation of the skull by setting up the lower threshold, ICHs were detected semi-automatically with a density threshold from 44 to 100 HU (Fig. 2A). HE was defined as relative hematoma growth > 33% or absolute hematoma growth > 6 mL compared with that on the baseline NCCT scan. The relative and absolute volume difference between the maximum volume within 72 h since the arrival and baseline volume of the hematoma was investigated.

### Statistical analysis

**Figure 3:**
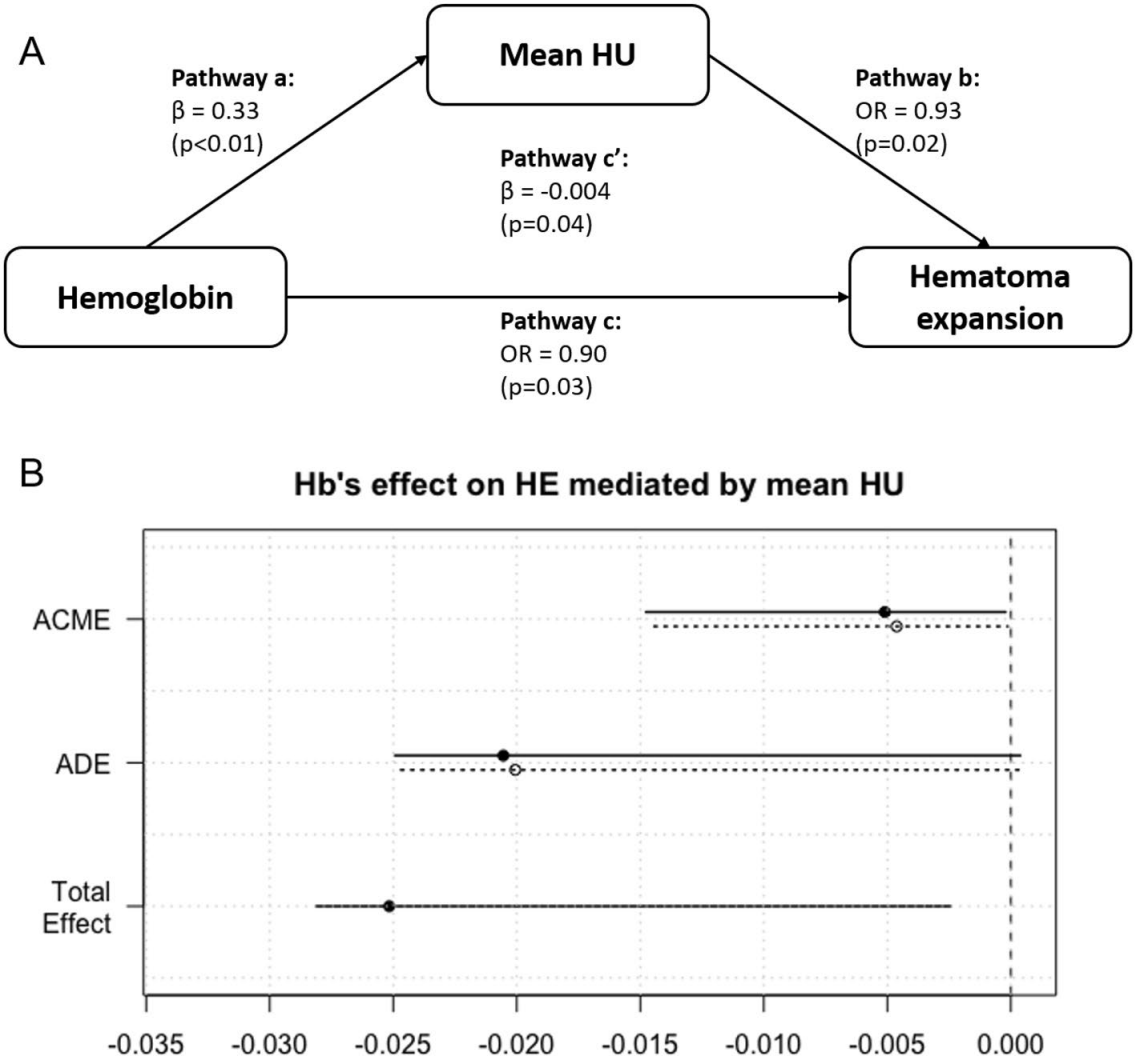
Mediation analysis of association between hemoglobin and HE with mediator mean HU. (**A**) Causal mediation effect of mean HU on the relationship between hemoglobin and hematoma expansion (HE). (**B**) Direct, indirect, and total effects of hemoglobin on HE mediated via the mean HU. HU, Hounsfield unit; β, coefficient; OR, odds ratio; ACME, average causal mediation effect; ADE, average direct effect.

The difference in clinical and imaging data between groups according to HE was analyzed using the χ^2^ test for categorical variables and t-test for continuous variables. Within- and between-rater reliabilities were analyzed using weighted kappa statistics. Mediation analysis was performed to confirm the association between hemoglobin levels and HE with respect to potential mediators, the mean HU of the hematoma. To affirm the requirements for mediation analysis defined by Baron and Kenny^12^, a linear regression model for continuous variables (pathway a in Fig. 3A) and binary probit regression model for dichotomous outcome variables (pathway b, c in Fig. 3A) were adopted. All pathways were tested using univariable and multivariable regression analyses, and unadjusted and adjusted estimated coefficient βs, odds ratios (OR), and 95% confidence intervals were reported. For multivariable regression analysis, age, sex, and previously known HE predictors (time from onset to CT, history of previous antiplatelet and anticoagulant agents, baseline hematoma volume) were selected as prespecified covariates for the first model^13^. The second model was additionally adjusted with baseline systolic blood pressure (> 140 mmHg or not) and location of hematoma (lobar vs. non-lobar) which were statistically significant in bivariate analysis. The relationships between volume difference and the mean HU and hemoglobin were examined using both univariable and multivariable regression analyses. We performed post-hoc subgroup analysis after excluding patients with previous antiplatelet agent and anticoagulant agent. The rate of HE and functional outcome according to tranexamic acid administration was also investigated.

Mediation analysis (pathway c′ in Fig. 3A) was performed with the algorithms proposed by Imai et al.^14^ The average causal mediation effect (ACME) and average direct effect (ADE) were estimated using nonparametric 1,000 bootstrapping. ACME stands for the change in probability for HE when the independent variable is held constant. Therefore, ACME represents the indirect effects of hemoglobin through the mediator, and ADE represents the direct effects of hemoglobin when the mediator is held constant^14^. Values of *p* < 0.05 were considered statistically significant. Statistical analyses were performed using R 3.6.2 (R Development Core Team, Vienna, Austria).

## Results

Among the 232 included patients, 80 (34.5%) experienced HE. Patients with HE were older, had higher baseline NIHSS scores, and were more likely to be on anticoagulants. Hemoglobin levels (Fig. 2B) and hematocrit were significantly higher in patients without HE than in patients with HE. In patients with HE, the median time from onset to HE detection was 33.6 [Interquartile range 14.2–52.2] hours. Patients with HE presented with lower systolic blood pressure, larger hematoma volume, and lower mean hematoma HU than patients without HE. Patients with HE had poor functional outcomes after three months (modified Rankin scale score: 4–6; Table 1).

**Table 1:** Characteristics according to hematoma expansion.

	Hematoma expansion		*p*-value
	No (n = 152)	Yes (n = 80)	
Age	62.2 ± 13.7	68.1 ± 13.6	< 0.01
Male	94 (61.8%)	51 (63.8%)	0.89
Hypertension	124 (81.6%)	69 (86.2%)	0.47
Diabetes mellitus	28 (18.4%)	22 (27.5%)	0.15
Hyperlipidemia	36 (23.7%)	21 (26.2%)	0.79
Atrial fibrillation	11 (7.2%)	10 (12.5%)	0.28
Previous antiplatelet use	31 (20.4%)	15 (18.8%)	0.90
Previous anticoagulant use	8 (5.3%)	11 (13.8%)	0.05
NIHSS score at admission	12 [8–17]	16 [10–22]	< 0.01
Onset to CT time (hours)	2.2 [1.3–4.5]	1.9 [1.2–4.9]	0.74
**Hematoma location**			< 0.01
Basal ganglia	72 (47.4%)	23 (28.8%)	
Thalamus	47 (30.9%)	12 (15.0%)	
Lobar	18 (11.8%)	29 (36.2%)	
Infratentorial	15 (9.9%)	16 (20.0%)	
Intraventricular hemorrhage	42 (27.6%)	22 (27.5%)	1.00
Mean HU of baseline hematoma (HU)	58.5 ± 3.3	56.8 ± 3.0	< 0.01
Baseline hematoma volume (mL)	14.1 ± 13.8	24.3 ± 24.0	< 0.01
Systolic BP (mmHg)	179.3 ± 34.0	168.5 ± 33.0	0.02
**Laboratory test**			
Hemoglobin (g/dL)	14.2 ± 1.8	13.3 ± 2.4	< 0.01
Hematocrit (%)	42.6 ± 4.9	40.1 ± 6.6	< 0.01
White blood cell count (10^6^/L)	8860 ± 3141	9059 ± 3588	0.66
Platelet count (10^9^/L)	216.4 ± 62.4	210.5 ± 59.9	0.49
Prothrombin time (INR)	1.1 ± 0.3	1.1 ± 0.7	0.11
Creatinine (mg/dL)	0.8 ± 0.3	0.9 ± 0.5	0.07
Serum glucose (mg/dL)	138.6 ± 50.6	140.1 ± 40.4	0.80
**Hemostatic treatment**			
Intravenous tranexamic acid	16 (10.5%)	16 (20.0%)	0.07
3-month mRS 4–6	58 (38.2%)	54 (67.5%)	< 0.01

NIHSS, National Institute of Health Stroke Scale; LNT, last known normal time; CT, computed tomography; HU, Hounsfield unit; BP, blood pressure; INR, international normalized ratio; mRS, modified Rankin Scale.

The serum level of hemoglobin was positively associated with the mean HU (β = 0.47, *p* < 0.01) in univariable analysis. After adjusting the covariates (onset to CT time, previous administration of antithrombotic agents, age, sex), the multivariable linear regression model showed a linear relationship between hemoglobin levels and HU (β = 0.35, *p* < 0.01; pathway a in Fig. 3A). The previously set potential mediator, the mean HU of the hematoma on the first CT scan was significantly associated with a higher possibility of HE in logistic regression analysis using the binomial probit model (unadjusted OR, 0.92; *p* < 0.01; adjusted OR, 0.93; *p* < 0.01; pathway b in Fig. 3A). These associations fulfill the requirements of the causal mediation relationship according to Baron and Kenny^12^.

In causal mediation analysis, significant direct and indirect effects were observed between hemoglobin levels and the possibility of HE, mediated through the mean HU in the multivariable model (pathway c′ in Fig. 3A). The data showed complete mediation via the mean HU of hematoma, as low hemoglobin increased the risk of HE indirectly through the mediator, the mean HU (ACME in Fig. 3B, *p* = 0.04). The direct effect of hemoglobin on HE was insignificant after the mediator was held constant (ADE in Fig. 3B, *p* = 0.07). Among the total effect of hemoglobin on HE, the proportion of indirect effect mediated via the mean HU was 19% (Table 2). However, the mediation effect of mean HU was not statistically significant in the model which was additionally adjusted for systolic blood pressure and location of hematoma (*p* = 0.08 for the total effect) and post-hoc subgroup analyses excluding patients with previous antiplatelet or anticoagulant use (Supplemental Table S1). Hematoma expansion was not different according to tranexamic acid use, in subgroups of patients with low hemoglobin level, low mean HU and previous antiplatelet or anticoagulant agent use (Supplemental Table S2).

**Table 2:** Association among hemoglobin levels, the mean HU and HE.

	Unadjusted		Adjusted (model 1*)		Adjusted (model 2**)	
	Value (95% CI)	*p*-value	Value (95% CI)	*p*-value	Value (95% CI)	*p*-value
**Indirect variable = hemoglobin, mediator = mean HU, direct variable = HE**						
Pathway a (β)	0.47 (0.27 to 0.66)	< 0.01	0.33 (0.12 to 0.54)	< 0.01	0.33 (0.11 to 0.54)	< 0.01
Pathway b (OR)	0.92 (0.87 to 0.97)	< 0.01	0.93 (0.88 to 0.99)	0.02	0.88 (0.88 to 0.94)	< 0.01
Pathway c (OR)	0.80 (0.69 to 0.91)	< 0.01	0.90 (0.81 to 0.99)	0.03	0.91 (0.82 to 0.99)	0.05
**Pathway c′**						
ACME	− 0.005 (− 0.017 to 0.000)	< 0.01	− 0.004 (− 0.014 to 0.000)	0.04	− 0.010 (− 0.025 to − 0.001)	0.01
ADE	− 0.014 (− 0.021 to − 0.001)	0.03	− 0.019 (− 0.025 to 0.001)	0.07	− 0.015 (− 0.022 to 0.017)	0.31
Total effect	− 0.019 (− 0.028 to − 0.002)	< 0.01	− 0.024 (− 0.028 to − 0.003)	0.01	− 0.026 (− 0.029 to 0.003)	0.08
Prop. mediated	0.27 (0.06 to 0.79)	< 0.01	0.19 (0.00 to 1.01)	0.05	0.40 (− 1.01 to 3.08)	0.09

*Model 1: Adjusted for age, sex, time from onset to CT, history of previous antiplatelet and anticoagulant agents, and baseline hematoma volume.

**Model 2: Model 1 + additionally adjusted for baseline systolic blood pressure and location of hematoma.

HU, Hounsfield unit; HE, hematoma expansion; CI, confidence interval; β, coefficient; OR, odds ratio; ACME, average causal mediation effect; ADE, average direct effect; Prop. mediated, proportion mediated.

For comparison, we examined the mediation effects of known NCCT markers of HE, including heterogeneous density, the black hole sign, blend sign, irregular shape, island sign, and satellite sign. The inter-rater reliability between the evaluations of the two stroke neurologists who inspected the NCCT markers was generally acceptable (Table 3). When the NCCT markers were assumed as mediators in the relationship between hemoglobin levels and HE, no mediation effects were observed (Supplemental Tables S3 and S4).

**Table 3:** NCCT predictive markers of HE.

	Total	Hematoma expansion			Kappa value
	(n = 232)	No (n = 152)	Yes (n = 80)	*p*-value	
**NCCT markers**					
Heterogeneous density	107 (46.1%)	59 (38.8%)	48 (60.0%)	0.03	0.74
Black hole sign	39 (16.8%)	21 (13.8%)	18 (22.5%)	0.14	0.84
Blend sign	45 (19.4%)	23 (15.1%)	22 (27.5%)	0.04	0.83
Irregular shape	112 (48.3%)	63 (41.4%)	49 (61.2%)	0.01	0.76
Island sign	95 (41.0%)	55 (36.2%)	40 (50.0%)	0.06	0.79
Satellite sign	119 (51.3%)	73 (48.0%)	46 (57.5%)	0.22	0.78

NCCT, non-contrast computed tomography; HE, hematoma expansion.

The mean HU also demonstrated an inverse relationship with relative difference between the baseline and maximum volume of the hematoma (β = − 0.09, *p* < 0.01, Supplementary Figure S1), and hemoglobin were related to both relative (β = − 0.08, *p* = 0.02) and absolute (β = -1.01, *p* < 0.01) change in the hematoma volume in univariable analysis. Multivariable analysis showed identical inverse relationships between the mean HU and relative change in the hematoma volume (β = − 0.08, *p* < 0.01) and between hemoglobin levels and the absolute change in the hematoma volume (β = − 0.77, *p* = 0.04).

## Discussion

Our study demonstrated the mediation effect of the mean HU of the hematoma on the inverse relationship between hemoglobin levels and HE under a limited set of covariates. In contrast, other NCCT predictors of HE did not mediate the association between hemoglobin levels and HE. The mean HU of the hematoma on the baseline NCCT scan was suggested to reflect the status of local hemostasis in the hyperacute phase of ICH^6^. The mediation effect of the mean HU may provide a mechanistic explanation that patients with low hemoglobin are prone to HE through impaired hemostasis.

ICH initially undergoes dynamic changes in the brain parenchyma and forms blood clots, where the density of the hematoma can reflect the time course of bleeding^15^. In the ultra-early stage of a hemorrhage, the fibrin fibrils and platelets build a meshwork-like plug, and red blood cells (RBCs) embed within the platelet–fibrin network to form a clot^16^. Subsequently, the clot contracts to obtain hemostasis, and during clot contraction, the serum is extruded and local concentration of hemoglobin increases^17,18^. CT attenuation of a hematoma increases during this dynamic phenomenon of hemostasis, and this increment is known to be majorly contributed by the protein component of hemoglobin^15,18^. One in-vitro CT study which analyzed the citrated blood revealed the linear relationship between hematocrit and attenuation of hematoma^19^. Another pioneering study also asserted that the absence of hyperdensity in acute hemorrhage might be related to anemia^15^.

Based on previous studies, we interpreted that ICH in patients with low hemoglobin level is more likely to be vulnerable to expand owing to insufficient clot contraction, which can be observed hypoattenuated on CT. Patients with anemia are known to have insufficient hemostasis because RBCs assist in the radial transportation of platelets toward the damaged vessel^16,20^. RBC can provide mechanical contractile force when the clot contracts^16^. RBCs can even transform into a polyhedral structure, which allows closer packing of the clot^5^. Therefore, patients with low hemoglobin in our study might have had HE owing to the failure of clot contraction and insufficient sealing effect of the clot, related to low RBC concentration within the hematoma. This hypothesis can also be supported by the association of an increased hematoma volume with the mean HU of hematoma in our data.

Our study findings may provide some insights into the current hemostatic strategies for patients with ICH. In previous clinical trials, hemostatic agents, such as recombinant activated factor VII and tranexamic acid, could limit hematoma growth but failed to improve the functional outcome^21,22^. These hemostatic agents may have led to a better functional outcome if they had been applied to patients with impaired local hemostasis, possibly owing to low hemoglobin concentration or coagulopathy. To the best of our knowledge, no proven strategies have been established to facilitate clot contraction and hemostasis. Contrary to expectations, platelet transfusion to reduce ICH growth in patients on antiplatelet therapy was shown to increase the risk of death compared with that following standard care in a multicenter, randomized trial^23^. However, as platelets play an important role in clot contraction, therapeutic strategies that promote clot contraction via actomyosin-driven platelet contraction or platelet–fibrin interaction in situ have the potential to be one of the novel hemostatic agents^24^.

Anemia is generally known to be associated with poor functional outcomes and mortality in ICH^3^. This association may be because anemia is simply a marker in advanced age or underlying morbidity itself^25^. However, patients with anemia usually present with a larger ICH volume, which can lead to a poor prognosis^3,26^. Moreover, a recent study has shown that the effect of low hemoglobin on poor functional outcomes is partially mediated via the occurrence of HE^4^. Brain tissue oxygenation can be compromised in critically ill patients with ICH^27^. In this regard, packed RBC transfusion has been attempted to improve the outcome in ICH, but the treatment effect was controversial^3,25^. The results of our study suggest that lower hemoglobin level may cause HE by delaying the maturation of hematoma^3,25^. Thus, if packed RBC transfusion is considered, it may be more appropriate in the hyperacute phase to prevent HE; it is unclear that packed RBC transfusion facilitates hemostasis in situ.

This study has several limitations. Because it was a single center study with a limited sample size. The mediation effects were not observed in the model adjusted for additional covariates and subgroup analyses excluding patients with prior antiplatelet or anticoagulant use. This result suggests the observed mediation effect was not robust enough and additional confounders can affect the relationship. Previous antiplatelet or anticoagulant agents and use of reversal or hemostatic agents, may affect the relationship between hemoglobin, mean HU and HE, although it could not be sufficiently evaluated in our study due to the small sample size. Therefore, our study is still hypothesis generating and the results should be validated in large and different population. In addition, only patients who were admitted to a neurology department were enrolled and inevitably had lower severity and a smaller hematoma size, resulting in selection bias. Given the weakness of a retrospective study, time and the number of follow-up imaging studies were inconsistent. Despite our efforts, unmeasured confounders might exist that might not have been included in the multivariable analyses, for instance, CT angiographic spot sign, volume of intraventricular hemorrhage and apolipoprotein E genotype.

## Conclusions

The mean HU of the hematoma on NCCT mediated the causal relationship between low hemoglobin levels and a high possibility of HE. This mediation effect suggests that low hemoglobin causes ineffective hemostasis, which is represented by the low density of the hematoma and results in its expansion. The mean HU of the hematoma may help identify future hemostatic therapies to prevent HE in patients with ICH.

## Supplementary Information

Supplementary Information.

## Data Availability

All relevant anonymized datasets presented within the article are available from the corresponding author on reasonable requests.
