# Supplementary material for: Mediation effects of mean Hounsfield unit on relationship between hemoglobin and expansion of intracerebral hemorrhage
Source: Sci Rep. 2021 Aug 26;11:17236. doi: 10.1038/s41598-021-96790-x (PMC8390671; doi:10.1038/s41598-021-96790-x)
Supplement: Supplementary file 1 — Supplementary Information. [file 41598_2021_96790_MOESM1_ESM.docx]

**Supplemental Materials:**

**Mediation Effects of Mean Hounsfield Unit on Relationship between Hemoglobin and Expansion of Intracerebral Hemorrhage**

Yong Soo Kim, MD, MSc^1^, Han-Gil Jeong, MD, MSc^1,2^, Hee-Yun Chae, MD, MSc^1,3^, Beom Joon Kim, MD, PhD^1^, Jihoon Kang, MD, PhD^1^, Jun Yup Kim, MD, MSc^1^, Tackeun Kim, MD, MSc^2^, Jae Seung Bang, MD^2^, Hee-Joon Bae, MD, PhD^1^, Chang Wan Oh, MD, PhD^2^, Moon-Ku Han, MD, PhD^1^

1. Department of Neurology, Seoul National University Bundang Hospital, Seongnam, Republic of Korea
2. Department of Neurosurgery, Seoul National University Bundang Hospital, Seongnam, Republic of Korea
3. Department of Neurology, Chungbuk National University Hospital, Cheongju, Republic of Korea

**Corresponding Author:**

Han-Gil Jeong

[han.g.jeong@gmail.com](mailto:han.g.jeong@gmail.com)

Department of Neurosurgery and Neurology, Seoul National University Bundang Hospital, 300 Gumi-dong, Bundang-gu, Seongnam, Gyeonggi-do, 463–707, Republic of Korea.

**Supplemental Table 1. Mediation analysis in subgroups excluding prior antiplatelet and/or anticoagulant agents use**

|  | Patients without history of  antithrombotic agents | | Patients without history of  antiplatelet agents | | Patients without history of  anticoagulant agents | |
| --- | --- | --- | --- | --- | --- | --- |
| Pathway | Crude OR (95% CI) | *p*-value | Crude OR (95% CI) | *p*-value | Crude OR (95% CI) | *p*-value |
| a (β) | 0.51 (0.26-0.75) | <0.01 | 0.55 (0.32-0.77) | <0.01 | 0.44 (0.24-0.65) | <0.01 |
| b (OR) | 0.90 (0.84-0.96) | <0.01 | 0.90 (0.84-0.95) | <0.01 | 0.92 (0.86-0.97) | <0.01 |
| c (OR) | 0.90 (0.81-1.00) | 0.05 | 0.87 (0.79-0.95) | <0.01 | 0.90 (0.82-0.98) | 0.02 |
| c’ |  |  |  |  |  |  |
| ACME | -0.014 (-0.030 to -0.001) | <0.01 | -0.006 (-0.024-0.000) | <0.01 | -0.009(-0.022 to -0.001) | <0.01 |
| ADE | -0.013 (-0.020-0.018) | 0.35 | -0.010 (-0.017-0.002) | 0.08 | -0.017 (-0.023-0.008) | 0.13 |
| Total effect | -0.019 (-0.029-0.000) | 0.05 | -0.016 (-0.027 to -0.001) | <0.01 | -0.027 (-0.030 to -0.003) | 0.02 |
| Prop. mediated | 0.51 (0.09-2.75) | 0.05 | 0.39 (0.15-1.06) | <0.01 | 0.35 (0.08-1.60) | 0.02 |
| Pathway | Adjusted OR (95% CI) | *p*-value | Adjusted OR (95% CI) | *p*-value | Adjusted OR (95% CI) | *p*-value |
| a (β) | 0.42 (0.16-0.69) | <0.01 | 0.45 (0.20-0.69) | <0.01 | 0.34 (0.11-0.57) | <0.01 |
| b (OR) | 0.82 (0.75-0.90) | <0.01 | 0.84 (0.77-0.90) | <0.01 | 0.86 (0.80-0.92) | <0.01 |
| c (OR) | 0.93 (0.82-1.05) | 0.28 | 0.90 (0.81-1.01) | 0.08 | 0.93 (0.84-1.03) | 0.19 |
| c’ |  |  |  |  |  |  |
| ACME | -0.024 (-0.039 to -0.001) | <0.01 | -0.018 (-0.035 to -0.002) | 0.02 | -0.016 (-0.029 to -0.002) | 0.01 |
| ADE | 0.000 (-0.015-0.029) | 0.98 | -0.008 (-0.016-0.026) | 0.62 | -0.007 (-0.021-0.021) | 0.68 |
| Total effect | -0.024 (-0.029-0.010) | 0.37 | -0.026 (-0.028-0.007) | 0.14 | -0.023 (-0.029-0.009) | 0.23 |
| Prop. mediated | 1.00 (-4.88-7.07) | 0.37 | 0.68 (-4.50-5.86) | 0.14 | 0.68 (-4.50-5.86) | 0.23 |

HU, Hounsfield unit; HE, hematoma expansion; CI, confidence interval; β, coefficient; OR, odds ratio; ACME, average causal mediation effect; ADE, average direct effect; Prop. mediated, proportion mediated

**Supplemental Table 2. Hematoma expansion and outcome according to tranexamic acid treatment in various subgroups.**

|  | **Tranexamic acid** | |  |
| --- | --- | --- | --- |
| **(A) Low hemoglobin level* (n=49)** | | | |
|  | No (n=45) | Yes (n=4) | *p*-value |
| Hematoma expansion | 24 (53.3%) | 3 (75.0%) | 0.76 |
| 3-months mRS 4-6 | 36 (80%) | 3 (75.0%) | 1.00 |
| **(B) Low mean HU** (n=58)** | | | |
|  | No (n=48) | Yes (n=10) | *p*-value |
| Hematoma expansion | 25 (52.1%) | 7 (70.0%) | 0.49 |
| 3-months mRS 4-6 | 32 (66.7%) | 4 (40.0%) | 0.22 |
| **(C) Previous antiplatelet agent use (n=46)** | | | |
|  | No (n=39) | Yes (n=7) | *p*-value |
| Hematoma expansion | 10 (25.6%) | 5 (71.4%) | 0.052 |
| 3-months mRS 4-6 | 25 (64.1%) | 3 (42.9%) | 0.52 |
| **(D) Previous anticoagulant agent use (n=19)** | | | |
|  | No (n=14) | Yes (n=5) | *p*-value |
| Hematoma expansion | 9 (64.3%) | 2 (40.0%) | 0.68 |
| 3-months mRS 4-6 | 10 (71.4%) | 3 (60.0%) | 1.00 |
| **Total (A – D) (n=116)** | | | |
|  | No (n=98) | Yes (n=18) | *p*-value |
| Hematoma expansion | 42 (42.9%) | 12 (66.7%) | 0.11 |
| 3-months mRS 4-6 | 64 (65.3%) | 9 (50.0%) | 0.33 |

*Low hemoglobin level refers to hemoglobin level < 13g/dL in male and < 12g/dL in female

**Mean HU under 1^st^ quartile (55.6 HU) was defined as low Mean HU

HU, Hounsfield unit; mRS, modified Rankin Scale

| **Supplemental Table 3. Mean HU, baseline hematoma volume, and hemoglobin according to the presence of NCCT markers** | | | | |
| --- | --- | --- | --- | --- |
|  | Heterogeneous density | |  |  |
|  | No (n=125) | Yes (n=107) | *p*-value |  |
| Mean HU | 58.0 ± 3.4 | 57.8 ± 3.1 | 0.70 |  |
| Baseline volume (mL) | 12.4 ± 14.8 | 23.7 ± 20.7 | <0.01 |  |
| Hemoglobin (g/dL) | 14.1 ± 2.0 | 13.7 ± 2.1 | 0.10 |  |
|  | Black hole sign | |  |  |
|  | No (n=193) | Yes (n=39) | *p*-value |  |
| Mean HU | 57.7 ± 3.3 | 59.0 ± 3.1 | 0.02 |  |
| Baseline volume (mL) | 15.2 ± 15.5 | 29.8 ± 26.6 | <0.01 |  |
| Hemoglobin (g/dL) | 13.9 ± 2.1 | 14.1 (±1.8) | 0.60 |  |
|  | Blend sign | |  |  |
|  | No (n=187) | Yes (n=45) | *p*-value |  |
| Mean HU | 57.8 ± 3.4 | 58.5 ± 2.8 | 0.18 |  |
| Baseline volume (mL) | 15.6 ± 17.2 | 26.1 ± 21.8 | <0.01 |  |
| Hemoglobin (g/dL) | 14.0 ± 2.1 | 13.7 ± 1.8 | 0.34 |  |
|  | Irregular shape | |  |  |
|  | No (n=120) | Yes (n=112) | *p*-value |  |
| Mean HU | 57.5 ± 3.4 | 58.4 ± 3.1 | 0.05 |  |
| Baseline volume (mL) | 10.6 ± 10.7 | 25.1 ± 22.1 | <0.01 |  |
| Hemoglobin (g/dL) | 14.2 ± 2.0 | 13.7 ± 2.1 | 0.07 |  |
|  | Island sign | |  |  |
|  | No (n=137) | Yes (n=95) | *p*-value |  |
| Mean HU | 57.8 ± 3.4 | 58.1 ± 3.1 | 0.55 |  |
| Baseline volume (mL) | 11.0 ± 10.6 | 27.2 ± 23.1 | <0.01 |  |
| Hemoglobin (g/dL) | 14.1 ± 2.0 | 13.6 ± 2.1 | 0.05 |  |
|  | Satellite sign | |  |  |
|  | No (n=113) | Yes (n=119) | *p*-value |  |
| Mean HU | 57.9 ± 3.4 | 58.0 ± 3.2 | 0.76 |  |
| Baseline volume (mL) | 11.3 ± 12.8 | 23.6 ± 21.2 | <0.01 |  |
| Hemoglobin (g/dL) | 14.1 ± 2.3 | 13.8 ± 1.9 | 0.32 |  |

HU, Hounsfield unit; NCCT, non-contrast computed tomography

| **Supplemental Table 4. Association among hemoglobin, NCCT markers, and HE** | | | | | | | |
| --- | --- | --- | --- | --- | --- | --- | --- |
|  | Value (95% CI) | *p*-value |  | | Value (95% CI) | *p*-value |  |
| IV=hemoglobin, mediator=heterogeneous density, DV=HE | | | | | | | |
| Unadjusted | | | | Adjusted | | | |
| Pathway a (OR) | 0.94 (0.86-1.01) | 0.10 | Pathway a (OR) | | 0.95 (0.87-1.05) | 0.35 |  |
| Pathway b (OR) | 1.61 (1.14-2.27) | <0.01 | Pathway b (OR) | | 1.32 (0.90-1.93) | 0.14 |  |
| Pathway c (OR) | 0.87 (0.80-0.95) | <0.01 | Pathway c (OR) | | 0.89 (0.81-0.98) | 0.02 |  |
| Pathway c’ |  |  | Pathway c’ | |  |  |  |
| ACME | -0.007 (-0.012-0.005) | 0.60 | ACME | | 0.003 (-0.009-0.005) | 0.75 |  |
| ADE | -0.019 (-0.026 to -0.003) | <0.01 | ADE | | -0.024 (-0.028-0.000) | 0.05 |  |
| Total effect | -0.025 (-0.034 to -0.003) | <0.01 | Total effect | | -0.021 (-0.032-0.000) | 0.05 |  |
| Prop. mediated | 0.25 (-0.35-0.39) | 0.60 | Prop. mediated | | -0.12 (-0.03-0.00) | 0.76 |  |
| IV=hemoglobin, mediator=black hole sign, DV=HE | | | | | | | |
| Unadjusted | | | | Adjusted | | | |
| Pathway a (OR) | 1.03 (0.93-1.13) | 0.59 | Pathway a (OR) | | 0.97 (0.86-1.10) | 0.62 |  |
| Pathway b (OR) | 1.49 (0.95-2.33) | 0.07 | Pathway b (OR) | | 1.22 (0.75-2.00) | 0.42 |  |
| Pathway c (OR) | 0.87 (0.80-0.95) | <0.01 | Pathway c (OR) | | 0.89 (0.81-0.98) | 0.02 |  |
| Pathway c’ |  |  | Pathway c’ | |  |  |  |
| ACME | -0.001 (-0.005-0.005) | 0.84 | ACME | | 0.000 (-0.007-0.004) | 0.87 |  |
| ADE | -0.020 (-0.029 to -0.003) | <0.01 | ADE | | -0.024 (-0.029-0.000) | 0.05 |  |
| Total effect | -0.021 (-0.030 to -0.003) | <0.01 | Total effect | | -0.025 (-0.030- to -0.001) | 0.04 |  |
| Prop. mediated | 0.06 (-0.26-0.20) | 0.85 | Prop. mediated | | 0.01 (-0.03-0.00) | 0.88 |  |
| IV=hemoglobin, mediator=blend sign, DV=HE | | | | | | | |
| Unadjusted | | | | Adjusted | | | |
| Pathway a (OR) | 0.96 (0.87-1.05) | 0.32 | Pathway a (OR) | | 0.94 (0.84-1.05) | 0.23 |  |
| Pathway b (OR) | 1.55 (1.02-2.35) | 0.04 | Pathway b (OR) | | 1.26 (0.80-1.98) | 0.31 |  |
| Pathway c (OR) | 0.87 (0.80-0.95) | <0.01 | Pathway c (OR) | | 0.89 (0.81-0.98) | 0.02 |  |
| Pathway c’ |  |  | Pathway c’ | |  |  |  |
| ACME | 0.000 (-0.010-0.005) | 1.00 | ACME | | -0.005 (-0.009-0.005) | 0.74 |  |
| ADE | -0.019 (-0.027 to -0.003) | <0.01 | ADE | | -0.024 (-0.028 to -0.002) | 0.04 |  |
| Total effect | -0.019 (-0.032 to -0.003) | <0.01 | Total effect | | -0.029 (-0.031 to -0.002) | 0.04 |  |
| Prop. mediated | 0.00 (-0.34-0.38) | 1.00 | Prop. mediated | | 0.17 (-0.36-0.47) | 0.73 |  |
| IV=hemoglobin, mediator=irregular shape, DV=HE | | | | | | | |
| Unadjusted | | | | Adjusted | | | |
| Pathway a (OR) | 0.93 (0.86-1.00) | 0.07 | Pathway a (OR) | | 0.95 (0.86-1.04) | 0.30 |  |
| Pathway b (OR) | 1.54 (1.09-2.17) | 0.01 | Pathway b (OR) | | 1.26 (0.87-1.84) | 0.22 |  |
| Pathway c (OR) | 0.87 (0.80-0.95) | <0.01 | Pathway c (OR) | | 0.89 (0.81-0.98) | 0.02 |  |
| Pathway c’ |  |  | Pathway c’ | |  |  |  |
| ACME | 0.000 (-0.010-0.005) | 1.00 | ACME | | 0.006 (-0.008-0.005) | 0.78 |  |
| ADE | -0.019 (-0.026 to -0.002) | <0.01 | ADE | | -0.024 (-0.028 to -0.002) | 0.03 |  |
| Total effect | -0.019 (-0.033 to -0.002) | 1.00 | Total effect | | -0.018 (-0.030 to -0.002) | 0.03 |  |
| Prop. mediated | 0.00 (-0.33-0.37) | 1.00 | Prop. mediated | | -0.36 (-0.35-0.37) | 0.77 |  |

NCCT, non-contrast computed tomography; HE, hematoma expansion; CI, confidence interval; IV, indirect variable; DV, direct variable; OR, odds ratio; ACME, average causal mediation effect; ADE, average direct effect; Prop. Mediated, proportion mediated

**Supplementary Figure 1. Maximum relative and absolute volume increment of hematoma according to mean HU**


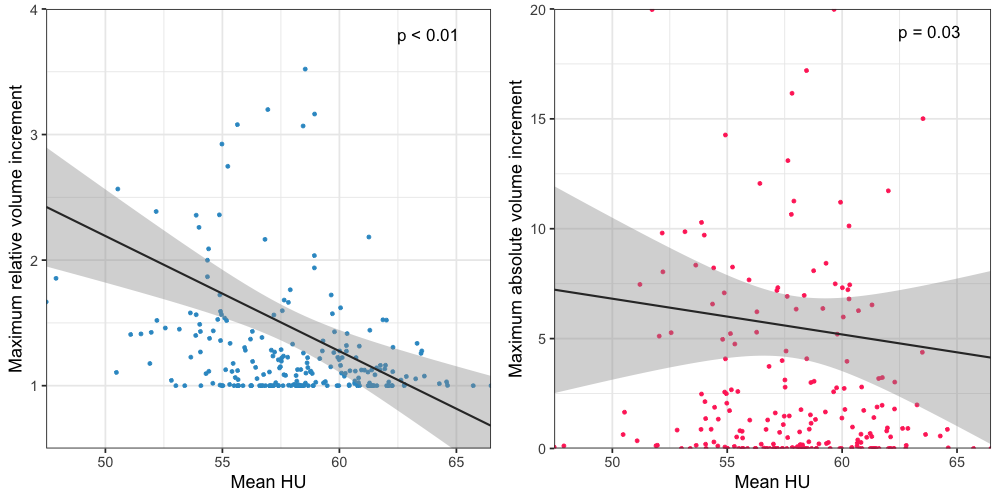


HU, Hounsfield unit
